# Anti-Fatigue Activity of Extracts of Stem Bark from *Acanthopanax senticosus*

**DOI:** 10.3390/molecules16010028

**Published:** 2010-12-24

**Authors:** Xue-Ling Zhang, Feng Ren, Wei Huang, Ren-Tao Ding, Qiu-Sheng Zhou, Xin-Wei Liu

**Affiliations:** 1 Physical Education College, Zhengzhou University, Zhengzhou 450044, China; E-Mails: zhangxllmu@gmail.com (X.-L.Z.); huangweicspr@163.com (W.H.); drt302@126.com (R.-T.D.); 2 Henan Ball Games Management Center, Zhengzhou 450044, China; E-Mail: 849599004@qq.com (Q-S.Z.); 3 Henan Research Institute of Sport Science, Zhengzhou, 450044, China; E-Mail: yizhou1963@163.com (X-W.L.)

**Keywords:** anti-fatigue, *Acanthopanax senticosus*, forced swimming test, mice

## Abstract

In the present study, we investigated the anti-fatigue activity in male Kunming mice of extracts of stem bark from *Acanthopanax senticosus* (ASSE) using a forced swimming test. Mice were divided into four groups (three ASSE administered groups and the control group). The control group were gavaged with distilled water and ASSE administered groups were gavaged with ASSE (100, 200 and 400 mg/kg). After four weeks, a forced swimming test was performed and the biochemical parameters related to fatigue were examined. The results suggested that ASSE could extend the swimming time to exhaustion of the mice, as well as increase the tissue glycogen contents, while decreasing the blood lactate and serum urea nitrogen contents. This indicated that ASSE had anti-fatigue activity and could elevate the exercise tolerance.

## 1. Introduction

*Acanthopanax senticosus* (also known as *Eleutherococcus senticosus* or Ciwujia, and previously known as Siberian ginseng) is an approximately two-meter high, hardy shrub native to the far eastern areas of the Russian taiga and the northern regions of China, Japan, and Korea [1]. It is known as an adaptogenic medicine, and it has been used as a crude drug to treat stress-induced physiological changes, various allergic conditions, inflammation and cancer [2,3,4,5,6]. The major active components of *Acanthopanax* senticosus include acanthoside, eleutheroside, chiisanoside, senticoside, triterpenic saponin, syringin, flavone, vitamin, minerals, β-sitosterol, sesamine and savinine [7,8,9]. Each of these chemical compounds is known to display diverse biological activities [10,11,12]. 

Fatigue is a complex phenomenon that can be described as a time-dependent exercise-induced reduction in the maximal force generating capacity of a muscle [13]. Alteration in performance tends to vary across sports that are influenced more or less by factors like decreased muscular power and endurance, decreased motor skill performance and mental lapses [14,15,16]. Since the available therapies for fatigue in modern medicine are very limited, potential alternatives from traditional medicine and their respective mechanisms of action is worth investigating [17]. Therefore, the present study is to investigate anti-fatigue activity of extracts of stem bark from *Acanthopanax senticosus* using a forced swimming test in male mice.

## 2. Results and Discussion

### 2.1. Effect of extracts of stem bark from Acanthopanax senticosus (ASSE) on the body weight of mice

**Figure 1:**
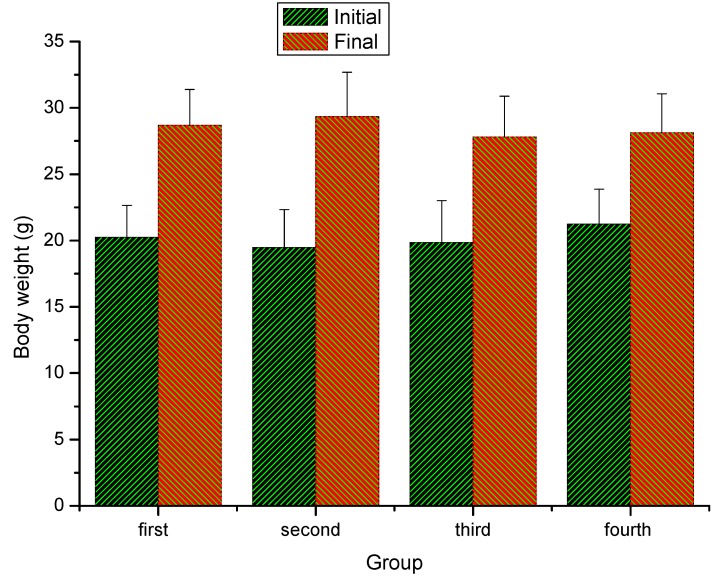
Effect of ASSE on the body weight of mice. Values represent the means ± SE (n = 30 per group).

The body weights of the mice were measured after they were administrated with different doses of ASSE for four weeks. As shown in Figure 1, the increased weights in the experimental groups were of no significant difference compared with the first (control) group (*P > 0.05*), so ASSE had no significant effect on the body weight of mice.

### 2.2. Effect of extracts of stem bark from Acanthopanax senticosus (ASSE) on swimming time to exhaustion of mice

**Figure 2:**
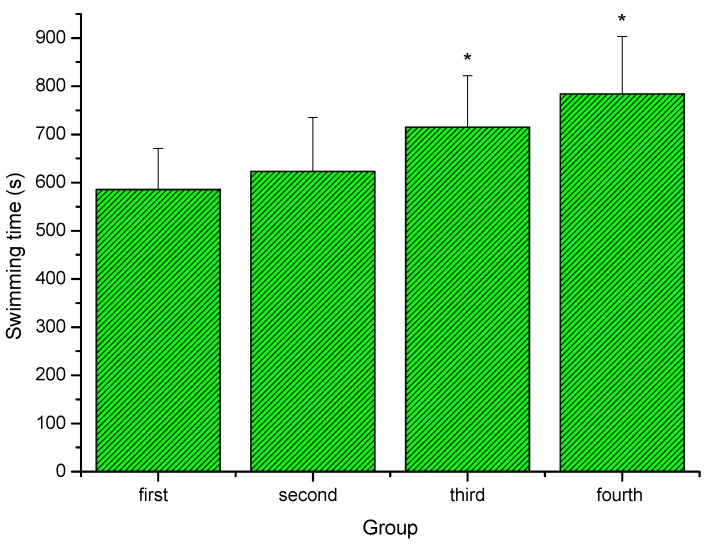
Effect of ASSE on swimming time to exhaustion of mice. Values represent the means ± SE (n = 10 per group). *P < 0.05 when compared to control group.

As shown in Figure 2, the third group (middle-dose) and fourth (high-dose )group showed a significant increase swimming time to exhaustion compared with the first (control) group (*P < 0.05*). However, swimming time in second (low-dose) group was longer than that of the first (control) group, but there was no significant difference (*P > 0.05*). The forced swimming test, which is perhaps one of the most commonly used animal models of behavioral despair, has been used extensively for the evaluation of the anti-fatigue properties of novel compounds [18,19,20,21,22]. To standardize the workload and reduce the swimming time, weights at specific body weight percentages were added to the chest or tail of the animal [23]. In the present study, the data showed that administration of ASSE could evidently extend swimming time to exhaustion of mice, which indicated that ASSE had anti-fatigue activity and could elevate the exercise tolerance.

### 2.3. Effect of extracts of stem bark from Acanthopanax senticosus (ASSE) on blood lactate of mice

**Figure 3:**
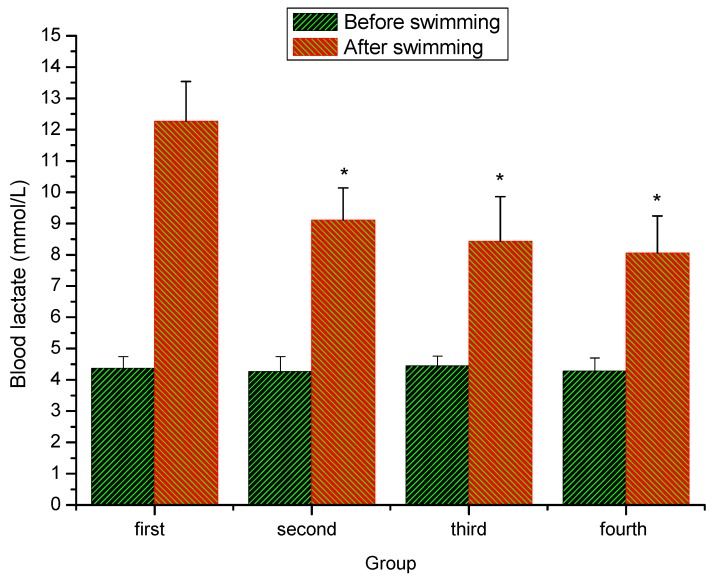
Effect of ASSE on blood lactate of mice.Values represent the means ± SE (n = 10 per group). *P < 0.05 when compared to control group.

As shown in Figure 3, there was no significant change in the blood lactate contents among all the groups before the swimming exercise. After swimming, blood lactate contents of the second (low-dose) group, the third group (middle-dose) and the fourth (high-dose) group were significantly lower than that of the first (control) group (*P < 0.05*). Previous studies have indicated that blood lactate is the glycolysis product of carbohydrate under anaerobic conditions, and glycolysis is the main energy source for intense exercise over a short time. The accumulation of blood lactate is a reason for fatigue during physical exercise [18,24,25,26,27], and rapid removal of lactate is beneficial to relieving fatigue. In the present study, the data indicated that ASSE could effectively delay the increase of lactate in the blood and postpone the appearance of physical fatigue.

### 2.4. Effect of extracts of stem bark from Acanthopanax senticosus (ASSE) on serum urea nitrogen (SUN) of mice

**Figure 4:**
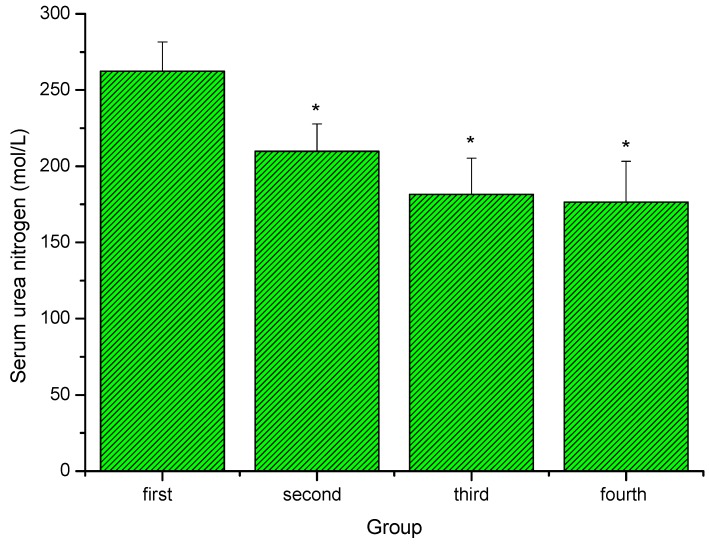
Effect of ASSE on serum urea nitrogen ofmice. Values represent the means ± SE (n = 10 per group). *P < 0.05 when compared to control group.

As shown in Figure 4, after swimming, serum urea nitrogen (SUN) contents of the second (low-dose) group, the third group (middle-dose) and the fourth (high-dose ) group were significantly lower than that of the first (control) group (*P < 0.05*). Serum urea nitrogen (SUN) is an important biochemical blood parameter related to fatigue. Urea is formed in the liver as the end product of protein-metabolism. During digestion, protein is broken down into amino acids. Amino acids contain nitrogen, which is removed as NH_4_^+^ (an ammonium ion), while the rest of the molecule is used to produce energy or other substances needed by the cell. There is a positive correlation between the urea nitrogen *in vivo* and the exercise tolerance [28,29,30]. In the present study, the data indicated that ASSE possessed the ability to lower or retard the formation of BUN after exercise.

### 2.5. Effect of extracts of stem bark from Acanthopanax senticosus (ASSE) on tissue glycogen of mice

As shown in Table 1, after swimming, liver and muscle glycogen contents of the second (low-dose) group, the third group (middle-dose) and the fourth (high-dose) group were significantly higher than that of the first (control) group (*P < 0.05*). Energy for exercise is derived initially from the breakdown of glycogen, after strenuous exercise muscle glycogen will be exhausted, and later, energy will form circulating glucose released by the liver [31]. Thus, the glycogen contents are sensitive parameters related to fatigue. In the present study, the data showed that ASSE might increase tissue glycogen contents of mice post exercise by improving glycogen reserve, or by reducing the glycogen consumption during exercise, or both. However, the detailed mechanism of this phenomenon is not clear and needs further study.

**Table 1:** Effect of ASSE on tissue glycogen of mice. Values represent the means ± SE (n = 10 per group). *P < 0.05 when compared to control group.

Group	Tissue glycogen (mg/g)	
	Liver	Muscle
first	7.81 ± 3.32	1.17 ± 0.34
second	14.29 ± 3.87*	1.81 ± 0.53*
third	17.22 ± 4.04*	2.06 ± 0.48*
fourth	18.86 ± 3.79*	1.97 ± 0.62*

## 3. Experimental

### 3.1. Chemicals

All chemicals were purchased from Zhengzhou Chemical Reagents Co., Ltd (Zhengzhou, China) unless otherwise indicated. Commercial diagnostic kits used to determine blood lactate, serum urea nitrogen (SUN) and tissue glycogen were purchased from Nanjing Jiancheng Bioengineering Institute (Nanjing, China).

### 3.2. Plant material and extraction

The authentic dry stem bark from *Acanthopanax senticosus* was obtained from a local herbal market (Zhengzhou, China). The plant has been authenticated and the voucher specimen has been deposited by Dr. Wang at the Herbarium of Zhengzhou University (Zhengzhou, China) under number ZZUC00156. The dry stem bark of *Acanthopanax senticosus* was cut into small pieces and extracted for 4 h at 70 °C with distilled water. Then the extracts were centrifuged at 1,000 × *g* for 15 min, and the supernatants were filtered through Whatman No.1 filter paper. The water extract of stem bark from *Acanthopanax senticosus* was named as ASSE.

### 3.3. Selection of animal and care

One hundred and twenty male Kunming mice weighing 18–22 g were used in the experiments. The animals were purchased from the Laboratory Animal Center, Medical College of Zhengzhou University (Zhengzhou, China.). They were group housed under the following laboratory conditions: temperature 22 ± 1 °C, humidity 40–60%, 12:12-L/D cycle, lights on at 07:00 h. Food and water were available *ad libitum*. All animals received humane care in compliance with the Henan Province Guidance on Experimental Animal Care. The protocol was approved by local animal study committee.

The mice were randomly divided into four groups (n = 30 in each group): The control group were administered 2.5 mL distilled water by gavage every day for four weeks. The low dose group were administered ASSE at 100 mg/kg body weight day for four weeks. The intermediate group were administered ASSE at 200 mg/kg body weight day for four weeks. The high-dose group were administered ASSE at 400 mg/kg body weight day for four weeks. ASSE was dissolved in 2.5 mL of distilled water. The doses of ASSE and 4-week treatment time used in this study were confirmed to be suitable and effective in tested mice, according to preliminary experiments.

### 3.4. Forced swimming test

The forced swimming test was used as described previously with some modifications [18,19,20,32,33,34,20,32]. After a period of four weeks, ten mice were taken out from each group for swimming exercise supporting constant loads (lead fish sinkers, attached to the tail) corresponding to 10% of their body weight. The swimming exercise was carried out in an acrylic plastic pool (50 cm × 50 cm × 40 cm) 30 cm deep with water maintained at 25 ± 2 °C. Exhaustion was determined by observing loss of coordinated movements and failure to return to the surface within 10 s [28,35,36,37], and the swimming time was immediately recorded.

### 3.5. Measurement of blood lactate contents of mice

After a period of four weeks, ten mice were taken out from each group for blood lactate analyses. The blood samples were collected from the veins of the tails of mice 30 mins after administration and 30 mins after weight loading swimming (2% body weight), respectively [29,38,39]. Then blood lactate contents were tested according to the recommended procedures provided by the commercial diagnostic kit.

### 3.6. Measurement of serum urea nitrogen and tissue glycogen contents of mice

After a period of four weeks, ten mice were taken out from each group for serum urea nitrogen (SUN) and tissue glycogen analyses. Mice were forced to swim for 90 mins without loads. After resting for an hour, the mice were killed to collect liver, gastrocnemius muscle and blood samples [24,25,40]. Serum urea nitrogen and tissue glycogen contents were tested according to the recommended procedures provided by the commercial diagnostic kit.

### 3.7. Statistical analysis

All values are presented as means ± SE. Statistical analysis was conducted by using unpaired t-tests or ANOVA and subsequently applying Tukey’s test (StatView: SAS Institute, Cary, NC). *P < 0.05* was considered statistically significant.

## 4. Conclusions

In conclusion, the data suggested that extracts of stem bark from *Acanthopanax senticosus* (ASSE) could extend the swimming time to exhaustion of the mice, as well as increase the tissue glycogen contents, and decrease the blood lactate and serum urea nitrogen contents. These results indicated that ASSE had anti-fatigue activity and could elevate exercise tolerance. However, further studies are necessary to clarify the detailed mechanism(s) involved in the anti-fatigue properties of ASSE.
